# Molecular changes in premenopausal oestrogen receptor-positive primary breast cancer in Vietnamese women after oophorectomy

**DOI:** 10.1038/s41523-017-0049-z

**Published:** 2017-11-27

**Authors:** Ben P. Haynes, Ophira Ginsburg, Qiong Gao, Elizabeth Folkerd, Maria Afentakis, Le Hong Quang, Pham Thi Han, Pham Hong Khoa, Nguyen Van Dinh, Ta Van To, Mark Clemons, Ian E. Smith, Mitch Dowsett

**Affiliations:** 1 The Ralph Lauren Centre for Breast Cancer Research, Royal Marsden Hospital, Fulham Road, London, UK; 2 Department of Medicine, University of Toronto, Toronto, Canada; 3 Department of Population Health, NYU School of Medicine/Laura and Isaac Perlmutter Cancer Center, NYU Langone Medical Center, New York, USA; 4 The Breast Cancer Now Toby Robins Research Centre, The Institute of Cancer Research, Fulham Road, London, UK; 5 Department of Breast Surgery, National Cancer Hospital, Hanoi, Vietnam; 6 Department of Pathology, National Cancer Hospital, Hanoi, Vietnam; 7 Department of Medicine, Division of Medical Oncology, The Ottawa Hospital and University of Ottawa, Ottawa, Canada; 8 The Breast Unit, Department of Medicine, Royal Marsden Hospital, Fulham Road, London, UK

## Abstract

For premenopausal women with primary ER + breast cancer, oophorectomy (OvX) is an evidence-based cost-effective option and is standard treatment in many countries. However, there is virtually no data describing the effects of OvX on breast tumour biology. We therefore, characterised the endocrine and genome-wide transcriptional impact of OvX in 56 premenopausal women with ER + breast cancer for 2 weeks prior to mastectomy. Plasma estradiol concentrations decreased from 406 ± 41 to 20.7 ± 2.6 pmol/l (mean ± sem) 24 h after OvX, and to 8.1 ± 0.8 pmol/l 2 weeks later at mastectomy. Ki67 decreased in 33/36 (91.7%) tumours. The expression of 655 genes changed significantly (FDR < 1%) with an absolute mean fold-change (FC) ≥ 1.25 (257 up, 398 down). Archetypal oestrogen-regulated genes (TFF1, GREB1, PGR and PDZK1) showed large decreases in expression (FC = 0.20–0.69; *p* < 1e-6-1e-7). Proliferation-associated genes (e.g. TOP2A, AURKA and UBE2C) were also strongly downregulated (FC = 0.38–0.56; *p* < 1e-7) along with putative progesterone-regulated genes (e.g. FKBP4, MYB; FC = 0.64–0.68; *p* < 1e-4-1e-7). The gene expression changes did not differ according to HER2 status and correlated strongly with the changes reported previously after aromatase inhibitor (AI) treatment in postmenopausal women (rho = 0.55, *p* < 1e-04). However, after OvX the mean FC was significantly higher compared to AI (*p* < 1e-04). In conclusion, changes in tumoural gene expression after OvX were largely similar, but of a greater magnitude to those observed after AI in postmenopausal patients; however, OvX appeared to have a greater effect on progesterone-regulated genes than AI.

## Genetics: Ovary removal alters gene activity in breast tumours

Surgical removal of the ovaries alters the expression of hundreds of genes in the tumour cells of premenopausal women with breast cancer. Ben Haynes from Royal Marsden Hospital in London, UK and colleagues characterised molecular changes in 56 premenopausal women from Vietnam with oestrogen receptor-positive breast cancer who underwent oophorectomies, a standard treatment for this patient population. They showed that blood levels of the hormone estradiol dropped precipitously following ovary-removal surgery. Levels of a protein that was indicative of tumour growth also went down, as did genes involved in regulating hormone signalling and cell proliferation. The results are consistent with those seen in postmenopausal women following treatment with an oestrogen-blocking drug, but oophorectomy had a more dramatic effect. The data could aid the search for predictive biomarkers of who stands to benefit most from ovary removal.

## Introduction

The use of surgical OvX was first described by Beatson as an endocrine treatment for breast cancer over 120 years ago.^1^ OvX with or without tamoxifen is an evidence-based cost-effective option for first-line adjuvant treatment of premenopausal women with ER + breast cancer after mastectomy who decline or otherwise lack access to chemotherapy.^2–4^ The majority of women with breast cancer live in low- or middle-income countries, where access to affordable, timely, evidence-based treatment options for breast cancer are often very limited. In higher-income countries, ovarian function suppression (OFS) is now most often achieved by so-called medical oophorectomy with gonadotrophin-releasing agonists (GnRHas), and has become an established treatment option for ER + breast cancer in premenopausal women most often in combination with tamoxifen.^4^ In the SOFT and TEXT trials, the combination of an aromatase inhibitor (AI) with OFS improved the 5-year breast cancer-free interval more than OFS plus tamoxifen, with the absolute difference in benefit ranging widely (1–15%) depending on the risk of recurrence.^5^ There was also a 5% benefit of tamoxifen plus OFS vs. tamoxifen alone in higher-risk patients. These trials have reinforced the application of OFS as adjuvant treatment for ER + premenopausal disease.

While this adjuvant therapy is clearly effective, in order to improve breast cancer outcomes associated with OvX, it is important to better understand the biological effects that OvX has in breast cancers, and to identify pre-treatment biomarkers that could allow it to be targeted to patients with the greatest potential to benefit. While we,^6–11^ and others,^12–16^ have acquired highly informative data relevant to patient management on the molecular effects of endocrine therapy on breast cancer in postmenopausal women, there is virtually no such information on the biological effects of OFS or OvX in premenopausal women.

Here we report unique data on whole-genome expression profiling of tumour biopsies before and after OvX in premenopausal women with ER + breast cancer undergoing this treatment for clinical management. We aimed to identify the most important genes and pathways associated with the response to OvX as well as determinants of that response. In addition, we aimed to assess the degree to which the relationships differed from those observed after an AI in postmenopausal women i.e. comparing withdrawal of oestrogen and progesterone vs. withdrawal of oestrogen alone.

## Results

**Fig. 1:**
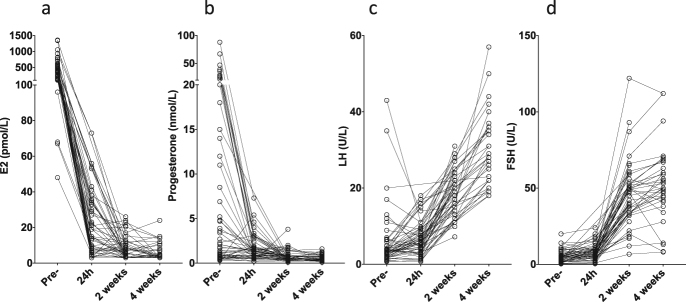
Changes in serum hormone concentrations of estradiol **a** progesterone **b** LH **c** and FSH **d** following OvX

Patient demographics are described in Supplementary Table 1. Ninety-three percent of patients were PgR + ve and thirty-eight percent were HER2 + ve. This was an unexpectedly high level of HER2 positivity, but was confirmed by repeating the IHC and FISH measurements for HER2; HER2 positivity correlated strongly with high levels of ERBB2 gene expression. Plasma oestradiol (E2) concentrations decreased from 406 ± 41 to 20.7 ± 2.6 pmol/l (mean ± sem) 24 h after OvX and to 8.1 ± 0.8 pmol/l by mastectomy (2 weeks), with no further significant decline at 4 weeks (6.6 ± 0.9 pmol/l) (Fig. 1a). All patients had values below 80 pmol/L after 24 h and below 30 pmol/L by 2 weeks after OvX. Progesterone levels showed a similar pattern to E2 with all values below 4 nmol/L by 2 weeks (and only one patient > 2nmol/L), while mean LH and FSH levels increased markedly after OvX (Fig. 1b–d). By 4 weeks all patients showed LH values > 18 IU/L, and all but 4 patients showed FSH values > 20 IU/L.

Paired FFPE tumour samples before and 2 weeks after OvX (time-points A or B and C, respectively) were available for 52 (A, B & C; *n* = 35, A & C; *n* = 17) of the 56 patients. Of the 122 FFPE samples, 18 were excluded from further analysis due to low cellularity (see consort diagram, Supplementary Fig. S1.) Paired frozen tumour samples were available for 53 (A, B & C; *n* = 42, A & C; *n* = 3, B & C; *n* = 8) of the 56 patients. Of the 136 frozen samples, 31 were excluded from further analysis due to low cellularity, and nine due to insufficient RNA yield/quality. RNA from the remaining 88 paired frozen samples (32 patients total; 24 with A, B & C samples; 5 with A & C and 3 with B & C) was arrayed on Illumina whole genome expression BeadChips (see consort diagram, Supplementary Figure 1). In cases where both A and B samples were available, a mean of the two results was used as the pre-treatment value for Ki67 (in FFPE samples) or gene expression.

**Fig. 2:**
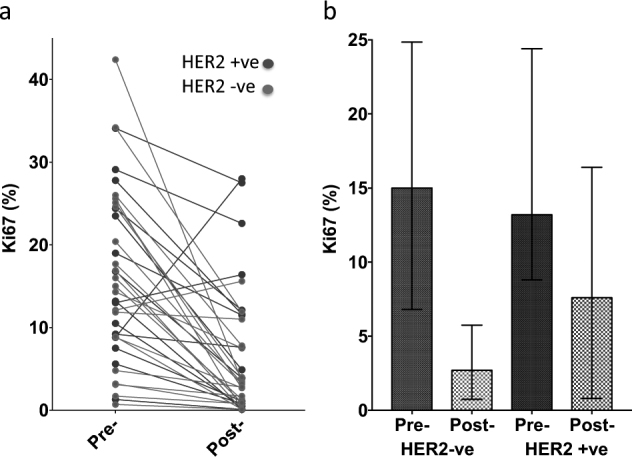
Changes in Ki67 in all patients **a** and in HER2 subgroups **b** after OvX. Mean ± IQR values are displayed

Ki67 expression by IHC decreased from a median of 14.7% (interquartile range; 8.8–24.6%) to 3.3% (0.8–11.4%) 2 weeks after OvX equating to a median 77.5% (IQR 42.6–90.8%) reduction in Ki67, but this varied greatly between patients (Fig. 2a). A reduction in Ki67 was seen in 33/36 (91.7%) of tumours, with a reduction of 50% in 25/36 (69.4%). A total of 16/36 (44.4%) of tumours reached complete cell cycle arrest^17^ (CCCA: Ki67 ≤ 2.7%). Baseline Ki67 was similar in the HER2 -ve (median 15.0%; IQR 6.8–24.9%) and HER2 + ve (13.2%; 8.8–24.4%) subgroups (Fig. 2b). The percentage fall in Ki67 after OvX was not significantly different (Mann–Whitney *p*-value = 0.23) between the HER2 -ve (median 81.9%, IQR 50.3–91.5%, 16/21 > 50% reduction; 11/21 (52.3%) attaining CCCA) and HER2 + ve subgroups (median 58.3%, IQR 19.4–85.7%, 9/15 > 50% reduction; 5/15 (33.3%) attaining CCCA). Of the three tumours that did not show a fall in Ki67, two were HER2 + ve, one of which showed a large rise in Ki67 (8.8 to 28%). There was a positive correlation between baseline ER score and the percentage decrease in Ki67 (Spearman rho = 0.38, *p* = 0.026).

**Fig. 3:**
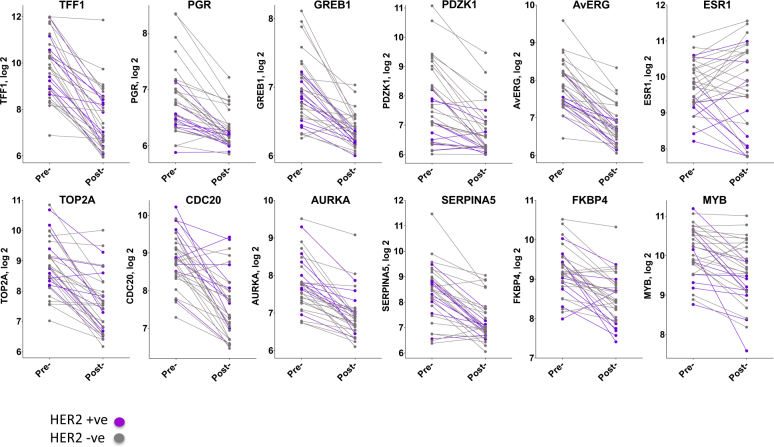
Change in expression of selected genes in individual patients after OvX

Class comparison analysis identified a total of 1361 genes (530 up, 831 down) that were significantly differentially expressed (FDR < 1%) after OvX. Of these, 655 genes changed with an absolute mean fold-change (FC) > 1.25 (257 up, 398 down; Supplementary Figure 2). TFF1 was the most highly downregulated gene (FC = 0.20; *p* < 1e-7). Other archetypal oestrogen-regulated genes (e.g. GREB1, PGR and PDZK1) also showed large decreases in expression (Fig. 3, Table 1). Proliferation-associated genes e.g. TOP2A, UBE2C and CDC20 were strongly downregulated (Fig. 3, Table 1). Putative progesterone-regulated genes e.g. FKBP5, MYB and SERPINA5 were also downregulated. Upregulated genes showed less consistency in function with the largest changes seen for many early-response genes (e.g. CYR61, DUSP1, FOSB and FOS) (Table 1). Eleven of the top 20 upregulated genes, including many of the early-response genes, were among the 31 upregulated genes that occurred in the POETIC presurgical 2-week window trial in the absence of drug-treatment,^18^ indicating that the change in expression of these genes is probably process-related and not treatment-related. The gene expression changes after OvX did not differ according to HER2 status, with no differences apparent between the HER2 +ve and −ve subgroups.

**Table 1:** Top up and downregulated genes after OvX and comparison to AI treatment^9^

		OvX				AI^9^	
Rank	Gene	All cases (*n* = 32)		HER2 −ve (*n* = 22)		All cases (*n* = 67)	
		Fold-change (post/pre)	95% CI	Fold-change (post/pre)	95% CI	Rank	Fold-change (post/pre)
Downregulated genes							
1	TFF1	0.20	0.15–0.27	0.21	0.14–0.31	1	0.34
2	UBE2C	0.38	0.31–0.47	0.38	0.30–0.49	2	0.42
3	SERPINA3	0.40	0.30–0.54	0.42	0.29–0.60	6	0.50
4	TOP2A	0.41	0.33–0.51	0.41	0.31–0.54	3	0.45
5	CDC20	0.43	0.34–0.54	0.43	0.33–0.55	7	0.51
6	NEK2	0.45	0.36–0.57	0.47	0.35–0.62	358	0.84
7	MSMB^1^	0.46	0.33–0.63	0.47	0.32–0.69	143	0.75
8	ASPM	0.47	0.39–0.56	0.46	0.37–0.59	17	0.56
9	SERPINA5	0.47	0.38–0.57	0.47	0.39–0.65	33	0.64
10	PRC1	0.47	0.40–0.55	0.46	0.36–0.57	12	0.53
11	NUSAP1	0.47	0.39–0.57	0.50	0.36–0.58	11	0.52
12	TFF3	0.48	0.37–0.62	0.47	0.33–0.64	129	0.75
13	PTTG1	0.48	0.40–0.58	0.48	0.37–0.59	26	0.63
14	TUBA3D	0.49	0.39–0.62	0.47	0.33–0.60	376	0.84
15	MAPT	0.49	0.42–0.57	0.45	0.40–0.56	452	0.85
16	NCAPG	0.50	0.43–0.59	0.50	0.42–0.60	25	0.62
17	KIAA0101	0.51	0.43–0.61	0.51	0.40–0.64	13	0.53
18	PDZK1	0.52	0.42–0.64	0.50	0.35–0.60	7	0.51
19	UHRF1	0.52	0.44–0.62	0.46	0.40–0.62	5	0.5
20	CELSR2	0.53	0.46–0.60	0.51	0.44–0.59	38	0.65
33	AURKA	0.56	0.49–0.64	0.58	0.49–0.68	24	0.61
38	BIRC5	0.57	0.50–0.65	0.58	0.50–0.67	889	0.93
52	E2F2	0.60	0.52–0.69	0.63	0.48–0.68	26	0.63
52	STC2^2^	0.60	0.47–0.77	0.57	0.49–0.81	19	0.58
75	FKBP4	0.64	0.54–0.74	0.68	0.59–0.79	128	0.75
105	GREB1	0.67	0.61–0.75	0.68	0.58–0.79	44	0.66
125	MYB^1^	0.68	0.58–0.80	0.75	0.64–0.88	19	0.58
131	PGR	0.69	0.62–0.76	0.66	0.57–0.75	105	0.74
Upregulated genes							
1	CYR61	3.47	2.63–4.58	3.13	2.22–4.40	–	–
2	DUSP1	3.40	2.49–4.64	3.65	2.48–5.39	–	–
3	FOSB^2^	2.60	1.59–4.23	2.66	1.39–5.09	–	–
4	FOS^1^	2.45	1.65–3.65	2.59	1.55–4.34	–	–
5	RGS1	2.38	1.87–3.04	2.50	1.90–3.28	–	–
6	CTGF	2.24	1.83–2.74	2.15	1.75–2.65	–	–
7	CPB1^2^	2.21	1.46–3.35	2.62	1.61–4.25	–	–
8	ZFP36	2.20	1.62–3.00	2.33	1.53–3.57	–	–
9	EGR1	2.17	1.60–2.94	2.31	1.55–3.42	–	–
10	ATF3^2^	2.07	1.46–2.95	2.23	1.36–3.64	–	–

Geometric mean fold-change values are shown, and the genes are ordered according to degree of change The top 20 genes downregulated by OvX plus selected additional downregulated genes of interest and the top 10 upregulated genes are shown All genes shown have univariate *P* < 1 × 10^−6^ with exception of ^1^ *P* < 1 × 10^−4^ and ^2^ *P* < 1 × 10^−3^

**Fig. 4:**
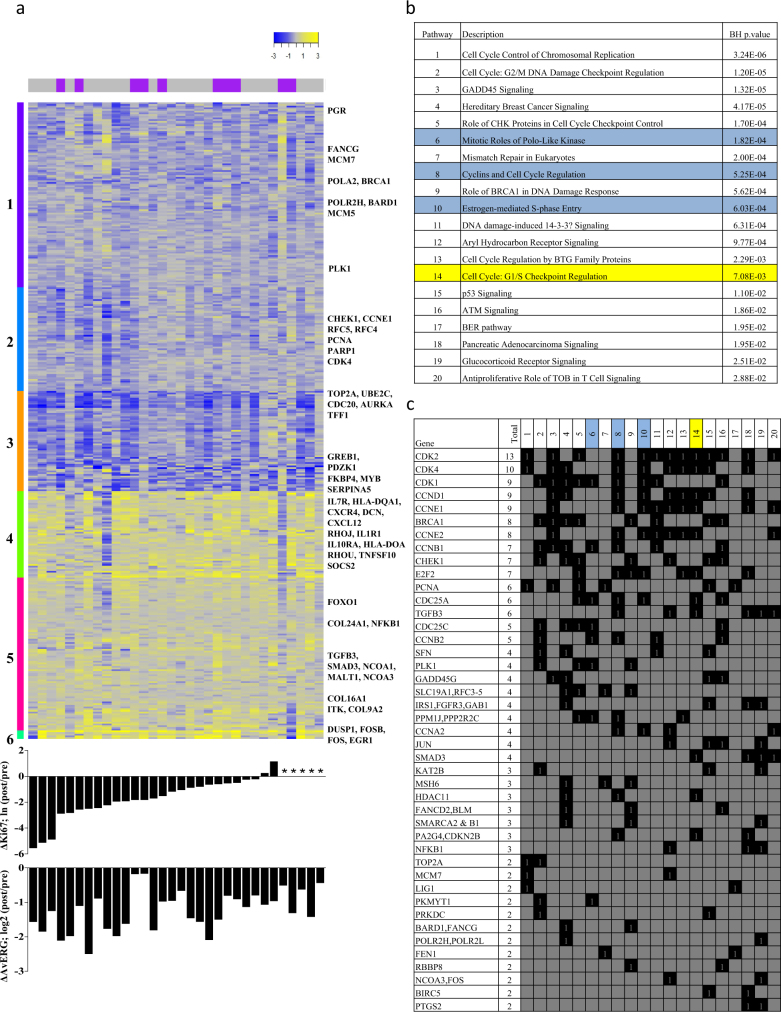
Heatmap of the values representing the change in expression of each of the 1361 significantly differentially expressed genes (FDR < 1%) with cases ordered by change in Ki67 (*indicates Ki67 data not available) **a**. Pathway analysis of the same 1361 genes showing the 20 pathways **b** and the genes within them **c** that were significantly overrepresented at adjusted *p*-values < 0.05. Significantly upregulated pathways are highlighted in blue and significantly downregulated pathways in yellow

One-way hierarchical clustering of the values representing the change in expression of each of the 1361 significantly differentially expressed genes in Fig. 4a shows the degree of heterogeneity in the transcriptional response. Most tumours showed strong downregulation of archetypal oestrogen-regulated genes (ERGs) and proliferation-associated genes (PAGs) (cluster 3). TFF1, AURKA and UBE2C were the only three genes that were downregulated in all cases. Clusters 1 and 2 comprised genes that were less consistently downregulated and included many cell cycle and DNA damage response pathway genes. The most highly upregulated genes including many of the early-response genes clustered very tightly in the small cluster 6 and showed consistent upregulation in 75–97% of the samples. Other strongly upregulated genes, including many immune-related genes (cytokines, chemokines) grouped in cluster 4. Genes in cluster 5, which were more variably upregulated, included collagens, cell surface molecules and additional immune-related genes (Fig. 4a). There were no clear patterns in the clustering according to HER2 status, change in Ki67 or change in the AvERG (average expression of PGR, GREB1, PGR and PDZK1^19^). There was a weak correlation between the changes in Ki67 and changes in AvERG (Spearman rho = 0.43 *p* = 0.026).

Pathway analysis (IPA) of the 1361 significantly differentially expressed genes was performed to identify overrepresented pathways (Fig. 4b). Twenty pathways were significant at adjusted *p*-values < 0.05, and the majority of these were proliferation-related. Cyclin-dependent kinases (CDK1, 2 and 4) and cyclins (CCNB1, CCND1 and CCNE1) were prominent in the majority of these pathways; 43 genes occurred in at least two pathways (Fig. 4c). Three pathways (‘Mitotic Roles of Polo-Like Kinase’, ‘Cyclins and Cell Cycle Regulation’ and ‘Oestrogen-mediated S-phase Entry’) were significantly inhibited, whilst ‘Cell Cycle: G1/S Checkpoint Regulation’ was significantly activated.

Higher baseline expression of ESR1 showed a weak statistically insignificant correlation with degree of decrease in the Ki67 (Spearman rho = −0.28, *p* = 0.15; Supplementary Figure 2a), but had stronger correlations with the degree of decrease in proliferation genes after OvX e.g. TOP2A (rho = −0.45, *p* = 0.0096; Supplementary Figure 3b), AURKA (rho = −0.36, *p* = 0.045) and CDC20 (rho = −0.31, *p* = 0.082). Baseline ESR1 expression also correlated with the change in AvERG (rho = −0.40, *p* = 0.025; Supplementary Figure 3c) and ER H-score (Spearman *r* = 0.452; *p* = 0.030). In contrast, baseline ERBB2 showed no correlation with change in Ki67, proliferation genes or AvERG after OvX.

We directly compared the gene expression changes observed after OvX with those following neoadjuvant AI treatment in ER + postmenopausal women with early breast cancer that we have previously reported.^9^ We found that changes in the overall gene expression after OvX strongly correlated (slope 0.55, Spearman rho = 0.55, *p* < 1e-04; Supplementary Figure 4A) with those reported after an AI.^9^ There were 432 genes (350 down, 82 up) whose expression was significantly affected by both OvX and AI (FDR < 1%). Seventy-six of these genes were affected to a significantly greater degree (*p* < 0.05) by OvX (58 down, 18 up) than AI, with an absolute FC 20% higher after OvX (geomean 1.44 vs. 1.20 for OvX and AI, respectively; *p* < 0.0001). Functional annotation of these 76 genes by IPA identified PGR as the top upstream regulator (*p* = 2.4e-07) with target molecules including FOXO1, IL1R1, PTGES and SERPINA5. Just one gene, NR4A2, was upregulated after OvX (FC 1.63) and downregulated after an AI (FC 0.77). No gene was affected more by AI than OvX. Five hundred and fifty-four genes (288 up, 266 down) significantly changed after OvX, but not after an AI. Pathway analysis of these genes revealed over-representation of genes associated with proliferation (e.g. CDK4, HDAC5 and SKP2) and immune response-related pathways (e.g. ATM, NFKB1 and SOCS2).

OvX had a significantly greater effect than AI on a proliferation metagene^20^ (203/229 genes; 16.7% reduction vs. 12.4% reduction; *p* = 0.039), but did not have a significantly greater effect than an AI on an ERG (oestrogen-regulated gene) metagene^9^ (31/34 genes; 32.2% reduction vs. 28.7%; *p* = 0.31).

We compiled a list of 245 putative progesterone-regulated genes based on literature reports^21–27^ (and Mohammed^21^ personal communication) and 144 of these genes were present in the final filtered data set (Supplementary Table 2). While changes in the expression of these genes after OvX were strongly correlated with the changes after AI in postmenopausal patients (slope 0.50, rho = 0.69, *p* < 1e-04; Supplementary Figure 4B), OvX had a greater effect than AI treatment both in terms of number of putative progesterone-regulated genes affected (50 vs. 41 with FDR < 5%) and the absolute mean FC (1.41 vs. 1.28, *p* = 0.020).

## Discussion

The use of OFS, either medically using GnRHas or surgically by OvX, as adjuvant treatment for ER + breast cancer in the premenopausal setting is well established. However, in contrast to studies of endocrine therapy in postmenopausal women, there is very little information describing the effects of OFS on breast tumour biology. In this study, we report unique data describing the endocrine and genome-wide transcriptional effects of OvX in premenopausal women with ER + breast cancer. Our objectives were to identify the most important genes and pathways associated with the response to OvX as well as determinants of that response and to evaluate the extent to which these differed from those observed after an AI in postmenopausal women. We found that after OvX, tumour cell proliferation was reduced in almost all patients and there were large decreases in ERGs, proliferation-associated genes and putative progesterone-regulated genes. The changes after OvX did not differ according to HER2 status and correlated strongly with those seen after AI treatment in postmenopausal women,^9^ but were of a greater magnitude. Moreover, OvX appeared to have more of an effect on progesterone-regulated genes than AI.

This pre-operative study provided a unique opportunity to investigate the endocrine and molecular effects of OvX in ER + premenopausal breast cancer and, to our knowledge, presents the only data describing the molecular effects of OvX on breast tumour biology. It should be noted that the large variation in hormone levels in premenopausal women that occurs through the menstrual cycle would lead to widely differing hormonal milieu at the time of OvX in this study. This could impact on the baseline molecular profiles since it has been shown that there are significant differences in the expression of ERGs and proliferation-associated genes through the menstrual cycle.^28,29^ In order to reduce the effect of such changes, we used the average Ki67 or gene expression value of two pre-treatment tumour samples taken 2 weeks apart as the baseline measurement in the majority of patients.

As expected, plasma hormone levels of E2 rapidly fell to reach postmenopausal levels 2 weeks after OvX due to removal of ovarian hormonal synthesis, with residual E2 now synthesised via aromatase in peripheral tissues as in postmenopausal women. Tumour cell proliferation measured by Ki67 showed large variability, and was similar to that seen after an AI in postmenopausal ER + ve breast cancer,^6,9^ with a reduction in the vast majority of tumours.

There is some evidence that HER2 + ve tumours do not respond as well as HER2 –ve tumours to AI therapy.^30^ In this Vietnamese ER + ve population there was a higher than expected level of HER2 positivity which was confirmed by gene expression measurements; although it is possible that this is due to ethnic origin it may also be due to chance. In contrast to previous reports^30^ we did not observe a higher baseline Ki67 in the HER2 + ve compared to the HER2 -ve patients, again this may be due to ethnic origin but it may also be due to chance. Whilst the reduction in Ki67 was nearly 1.5-fold greater in the HER2 –ve group compared to the HER2 + ve, this did not reach significance due to the large variability in Ki67 response and low numbers, such that the study had only 50% power to detect such a change as significant. Similarly, the gene expression changes after OvX did not differ according to HER2 status nor were there clear patterns in the clustering of the gene expression data according to HER2 status. Overall, our data suggest that HER2 positivity may not be a major determinant of response to OvX; this merits study in available large clinical trial cohorts.

The changes in gene expression after OvX mirrored those previously reported after an AI in postmenopausal ER + ve breast cancer^9^ and displayed strong downregulation of ERGs and PAGs and upregulation of immune-related genes and collagens with a high degree of heterogeneity. The most highly upregulated genes (e.g. CYR61, DUSP1, FOSB and FOS), which increased in almost all tumours, were early-response genes. However, the upregulation of these genes may be process- rather than treatment-related, as a very similar group of genes were upregulated in a study of the heterogeneity of gene expression in the absence of drug-treatment and this was attributed to ischaemia after surgery rather than any treatment effect.^18^

Direct comparison of the effects after OvX and AI therapy showed that whilst changes in overall gene expression were strongly correlated, the changes after OvX were of a greater magnitude. This could be because, in addition to suppressing E2 levels, OvX also suppresses progesterone levels, which does not occur after AI. This also leads to the hypothesis that OvX may affect progesterone-regulated genes to a greater extent than AI. Indeed, we found some evidence for this with PGR being the most highly significant upstream regulator of the genes affected more by OvX than AI; but overall, the differences between the two treatments were not large. This may reflect the fact that after an AI, the progesterone receptor gene (PGR) itself is downregulated, which would reduce progesterone signalling and receptor-mediated effects indirectly, despite progesterone levels (like E2 levels) being much lower in postmenopausal women.

OFS is now most often achieved by so-called medical oophorectomy with GnRHas. The changes seen in premenopausal ER + breast cancer in the current study using OvX to accomplish OFS would be expected to be similar to those seen after use of GnRHa alone after their initial stimulatory phase. However, over the first few months, ovarian oestrogen synthesis often shows partial recovery after a GnRHa as a result of a recovery of FSH levels.^31^ Thus, molecular changes with GnRHas may be of a lower magnitude than with OvX. It should be noted that the addition of an AI to GnRHa does not uniformly suppress the residual ovarian synthesis, and in some cases may lead to increased synthesis.^32,33^

In conclusion, we report for the first time the most important genes and pathways associated with the response to OvX. The degree of change in gene expression varied between patients and may reflect the degree of benefit derived from OvX, but this would need to be confirmed in much larger studies. The changes after OvX were largely similar, but of a greater magnitude to those after AI in postmenopausal patients; however, OvX appeared to have a greater effect on progesterone-regulated genes than AI.

## Methods

### Patients and study design

**Fig. 5:**
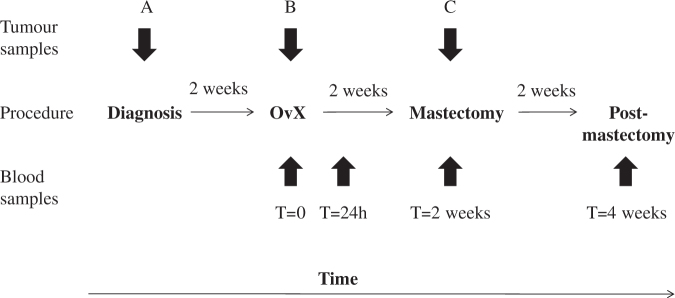
Study design and sampling schedule

A single-arm study of neoadjuvant OvX was conducted in 56 premenopausal women with ER + breast cancer in Vietnam. The primary objective of the study was to determine whether the variation in gene expression in different phases of the menstrual cycle could predict for change in Ki67 following oophorectomy. Thus, Ki67 was the primary end-point of the study, although the work reported here was on the conduct of a secondary objective. The study was planned to recruit 70 patients, but was curtailed at 56 because of recruitment difficulties.  The study design and sampling schedule are shown in Fig. 5. Premenopausal women with operable, palpable stage IIa-IIIb ER + invasive breast cancer for whom modified radical mastectomy and surgical bilateral salpingo-oophorectomy was planned as part of their breast cancer treatment were eligible.   Patients had to report regular menstrual cycles (≥three) of 25–35 days and must not have received any prior chemotherapy or radiotherapy for their cancer. Exclusion criteria included: metastatic disease, pregnancy, lactation within last 3 months, use of oral contraceptives or other hormonal contraceptives and concomitant use of medications known to influence oestrogen levels.

The study was approved by the Institutional Ethics Committee of the National Cancer Hospital, Hanoi, Vietnam, from where all study participants were recruited and by the Research Ethics Board of the University of Toronto, Canada, from where the study was coordinated. The Committee for Clinical Research at the Royal Marsden Hospital, London further approved the analysis of the samples collected in this trial. All participants provided written informed consent.

Blood samples were taken pre-OvX (on day of OvX prior to anaesthesia or pre-operative medication), 24 h post-OvX, 2 weeks post-OvX (pre-operative mastectomy) and 4 weeks post-OvX (Fig. 1). Breast tumour core biopsies were taken at three time-points; diagnosis (A), intra-operative at OvX (B; 2 weeks) and during mastectomy (C; 4 weeks). At each time-point one core biopsy was snap-frozen in liquid nitrogen for RNA extraction and another fixed in neutral buffered formalin and paraffin embedded for immunohistochemistry.

### Serum hormone measurements

Serum concentrations of E2 were measured by radioimmunoassay following pre-assay purification using an organic extraction as described previously.^34,35^ Progesterone was measured using a solid-phase radioimmunoassay (Beckman Coulter IM1188). LH and FSH were measured using immunoradiometric assays (IBL International MG12151 and Diasource KIP0841 respectively).

### Immunohistochemistry

Hematoxylin and eosin sections were prepared for all FFPE and frozen tumour samples, and were reviewed to confirm diagnosis and assess tumour content. Samples with tumour content < 40% were excluded from further analysis. Immunohistochemistry (IHC) and scoring for ER, PgR and Ki67 were performed as reported previously.^7,36^ HER-2 was measured immunohistochemically using the HercepTest (DakoCytomation) and by fluorescent in situ hybridisation (Vysis Pathvysion, Downers Grove, IL) according to manufacturer’s instructions. HER-2 was considered positive if immunohistochemical staining was scored 3 +, or 2 + if the fluorescence in situ hybridisation analysis indicated an amplification ratio of >2.0.

### Measurement of gene expression

Total RNA was extracted from frozen tissue using the RNeasy Mini kit (Qiagen, Sussex, UK). RNA quality was checked using an Agilent Bioanalyser (Santa Clara, CA, USA). Samples were excluded from further analysis if RNA quality was inadequate (RNA integrity values (RIN) of < 4.0). RNA amplification, labelling and hybridisation on HumanHT-12_v4 Expression BeadChips (Illumina, San Diego, CA, USA) were performed according to the manufacturer’s instructions. Raw data were extracted using GenomeStudio software, filtered to remove any non-expressed probes (detection *p*-value > 0.01) and transformed and normalised using variance-stabilising transformation and the robust spline normalisation method included in the lumi R package (http://www.bioconductor.org). Probes were further removed if they were not detected (*p* > 0.01) in > = 25% of samples resulting in 42.7% (20,216/47,323) of the starting probes remaining for the downstream data analysis.

To compare the gene expression changes observed after OvX in the current study with those following AI treatment, we used Illumina data (on HumanWG6_v2 Expression BeadChips) from our previously published neoadjuvant study of the effects of 2 weeks’ anastrozole monotherapy in 112 ER + postmenopausal women with early breast cancer.^9^ The individual Illumina data sets from the two studies were merged using nuID’s created from the Illumina probe sequences, yielding 29563 common probes. The combined data set was pre-processed as described above and the data were then batch corrected using the ComBat function in the sva R package (http://www.bioconductor.org). Probes were filtered out if they were not detected (*p* > 0.01) in > = 25% of samples resulting in 12716 of the probes remaining for the downstream data analysis.

### Data analysis and statistical methods

When multiple probes were mapped to the same gene, the most variable probe measured by interquartile range IQR across samples was selected to represent the gene. Multivariate permutation tests were used to identify differentially expressed genes between the paired samples. FDR values were calculated to allow for 80% probability that genes attained the set FDR threshold. The significantly differentially expressed genes were subjected to Ingenuity Pathway Analysis (IPA). The pathways were considered as significantly altered if the adjusted *p* < 0.05 after using Benjamini–Hochberg Multiple Testing Correction. Wilcoxon matched-pairs signed rank test was used to evaluate the significance of the percentage change of expression between pairs of samples. Power calculations indicated that there would be at least 85% power to detect correlations of greater than 0.6 between changes in gene expression and Ki67. The Mann–Whitney test was used to test for significance of any differences in the expression of genes after OvX compared to an AI. Wilcoxon matched-pairs signed rank test was used to evaluate the significance of differences in the absolute fold-change of individual genes after OvX compared to an AI. GraphPad Prism 6 (Graphpad Software Inc.) was used for some of the statistical analyses in this study. The reported *p*-values are two-tailed, with *p* < 0.05 considered as significant.

### Data availability

Gene expression data from this study is deposited at GEO with accession number GSE97221.

## Electronic supplementary material

Supplementary table 1

Supplementary table 2

Supplementary figure 1

Supplementary figure 2

Supplementary figure 3

Supplementary figure 4
