# Supplementary material for: Molecular changes in premenopausal oestrogen receptor-positive primary breast cancer in Vietnamese women after oophorectomy
Source: NPJ Breast Cancer. 2017 Nov 27;3:47. doi: 10.1038/s41523-017-0049-z (PMC5703856; doi:10.1038/s41523-017-0049-z)
Supplement: Supplementary file 1 — Supplementary table 1 [file 41523_2017_49_MOESM1_ESM.docx]

Supplementary table 1: demographic data

|  | All patients (n=56) | Patients with array data (n=32) |
| --- | --- | --- |
| **Age in years** |  |  |
| Median (range) | 42 (31-49) | 42 (31-49) |
| <35 | 7 (12.5%) | 5 (15.6%) |
| 35-39 | 12 (21.4%) | 7 (21.9%) |
| 40-44 | 17 (30.3%) | 9 (28.1%) |
| >=45 | 20 (35.7%) | 11 (34.4%) |
|  |  |  |
| **Lymph node status;** |  |  |
| 0 positive nodes | 29 (51.8%) | 17 (53.1%) |
| 1-3 positive nodes | 14 (25.0%) | 10 (31.3%) |
| > 3 positive nodes | 4 (7.1%) | 3 (9.4%) |
| Not known | 9 (16.1%) | 2 (6.3) |
|  |  |  |
| **Tumour grade;** |  |  |
| 1 | 8 (14.3%) | 6 (18.8%) |
| 2 | 42 (75.0%) | 25 (78.1%) |
| 3 | 1 (1.8%) | 1 (3.1%) |
| Not known | 5 (8.9%) | - |
|  |  |  |
| **Tumour size;** |  |  |
| Median (range) | 3.0 (0.5-4.0) | 3.0 (0.5-4.0) |
|  |  |  |
| **Tumour type** |  |  |
| Invasive ductal | 44 (78.6%) | 28 (87.5%) |
| Lobular | 2 (3.6%) | 1 (3.1%) |
| Mixed | 5 (8.9%) | 3 (9.4%) |
| Not known | 5 (8.9%) | - |
|  |  |  |
| **ER (all +ve)** |  |  |
| H-score; median (range) | 87 (1-165) | 105 (1-163) |
|  |  |  |
| **PR** |  |  |
| H-score; median (range) | 78 (0-190) | 61 (0-180) |
| -ve | 4 (7.1%) | 3 (9.4%) |
| +ve | 52 (92.9%) | 29 (90.6%) |
|  |  |  |
| **HER2** |  |  |
| -ve | 35 (62.5%) | 22 (68.8%) |
| +ve | 21 (37.5%) | 10 (31.2%) |
