# Supplementary material for: Molecular changes in premenopausal oestrogen receptor-positive primary breast cancer in Vietnamese women after oophorectomy
Source: NPJ Breast Cancer. 2017 Nov 27;3:47. doi: 10.1038/s41523-017-0049-z (PMC5703856; doi:10.1038/s41523-017-0049-z)
Supplement: Supplementary file 3 — Supplementary figure 1 [file 41523_2017_49_MOESM3_ESM.pptx]

## Slide 1
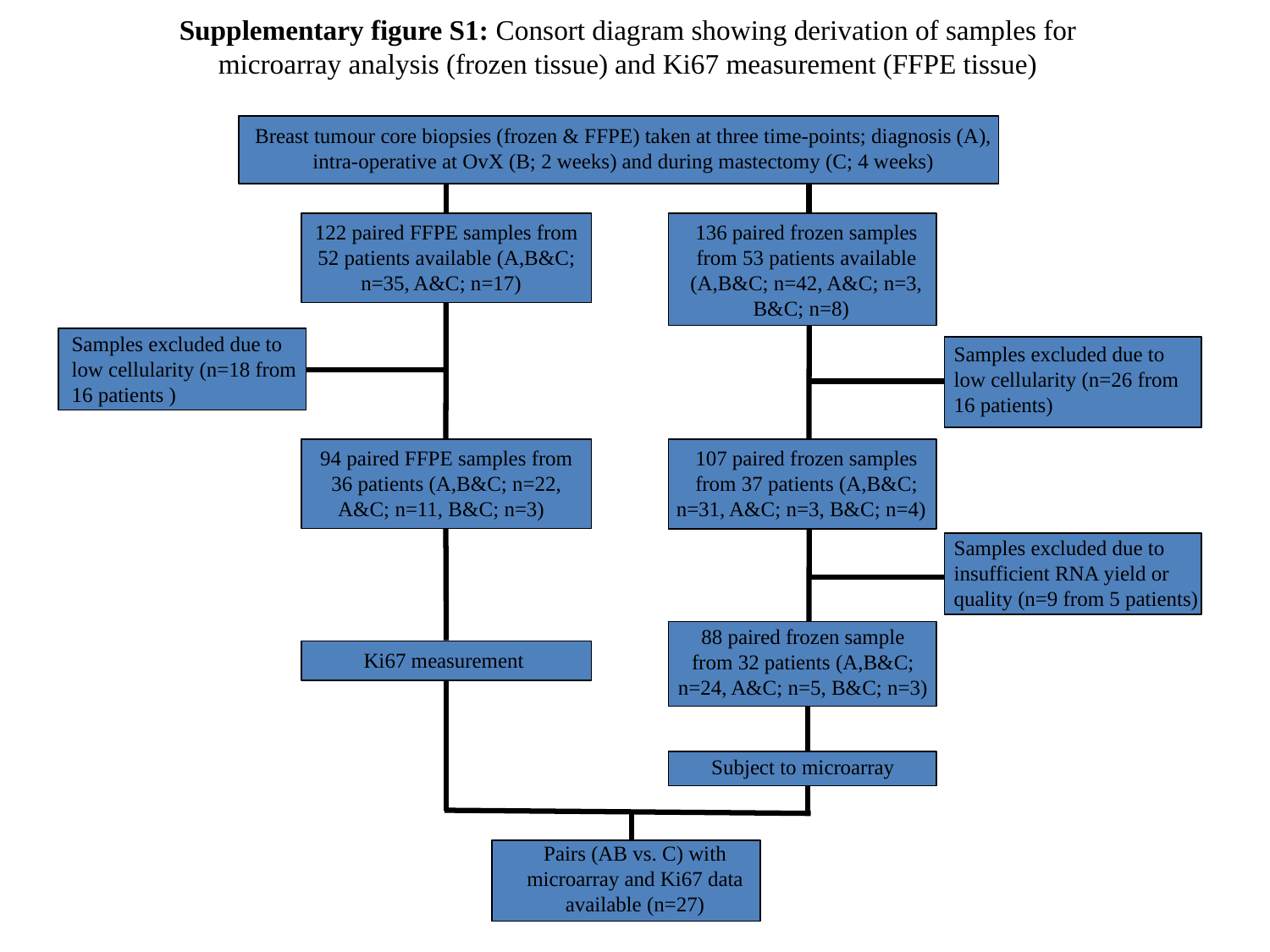

Supplementary figure S1: Consort diagram showing derivation of samples for microarray analysis (frozen tissue) and Ki67 measurement (FFPE tissue)
Breast tumour core biopsies (frozen & FFPE) taken at three time-points; diagnosis (A), intra-operative at OvX (B; 2 weeks) and during mastectomy (C; 4 weeks)
122 paired FFPE samples from 52 patients available (A,B&C; n=35, A&C; n=17)
136 paired frozen samples from 53 patients available (A,B&C; n=42, A&C; n=3, B&C; n=8)
Samples excluded due to low cellularity (n=18 from 16 patients )
Samples excluded due to low cellularity (n=26 from 16 patients)
94 paired FFPE samples from 36 patients (A,B&C; n=22, A&C; n=11, B&C; n=3)
107 paired frozen samples from 37 patients (A,B&C; n=31, A&C; n=3, B&C; n=4)
Samples excluded due to insufficient RNA yield or quality (n=9 from 5 patients)
88 paired frozen sample
from 32 patients (A,B&C; n=24, A&C; n=5, B&C; n=3)
Ki67 measurement
Subject to microarray
Pairs (AB vs. C) with microarray and Ki67 data available (n=27)
