# Supplementary material for: Molecular changes in premenopausal oestrogen receptor-positive primary breast cancer in Vietnamese women after oophorectomy
Source: NPJ Breast Cancer. 2017 Nov 27;3:47. doi: 10.1038/s41523-017-0049-z (PMC5703856; doi:10.1038/s41523-017-0049-z)
Supplement: Supplementary file 4 — Supplementary figure 2 [file 41523_2017_49_MOESM4_ESM.pptx]

## Slide 1
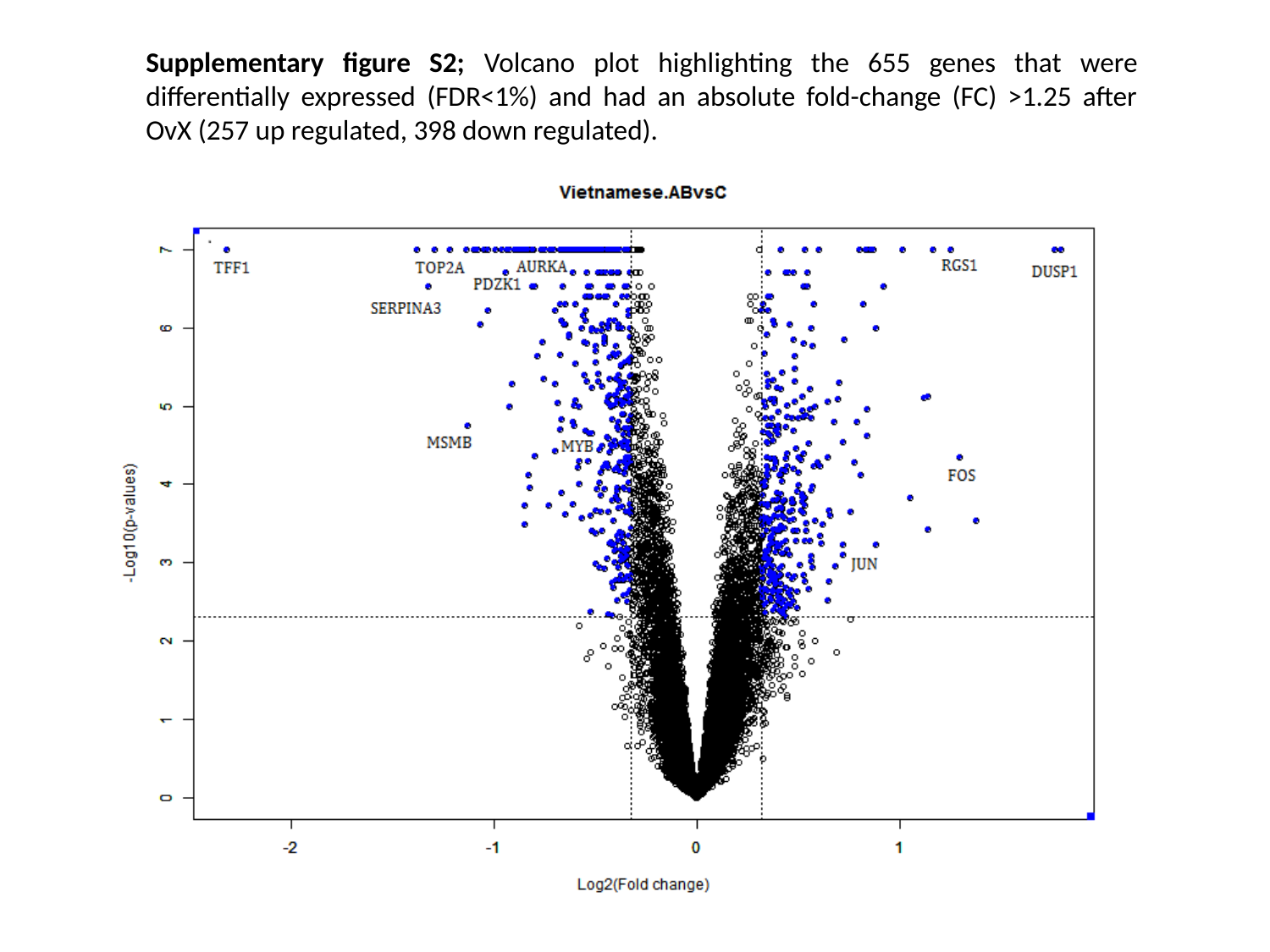

Supplementary figure S2; Volcano plot highlighting the 655 genes that were differentially expressed (FDR<1%) and had an absolute fold-change (FC) >1.25 after OvX (257 up regulated, 398 down regulated).
