# Supplementary material for: Molecular changes in premenopausal oestrogen receptor-positive primary breast cancer in Vietnamese women after oophorectomy
Source: NPJ Breast Cancer. 2017 Nov 27;3:47. doi: 10.1038/s41523-017-0049-z (PMC5703856; doi:10.1038/s41523-017-0049-z)
Supplement: Supplementary file 5 — Supplementary figure 3 [file 41523_2017_49_MOESM5_ESM.pptx]

## Slide 1
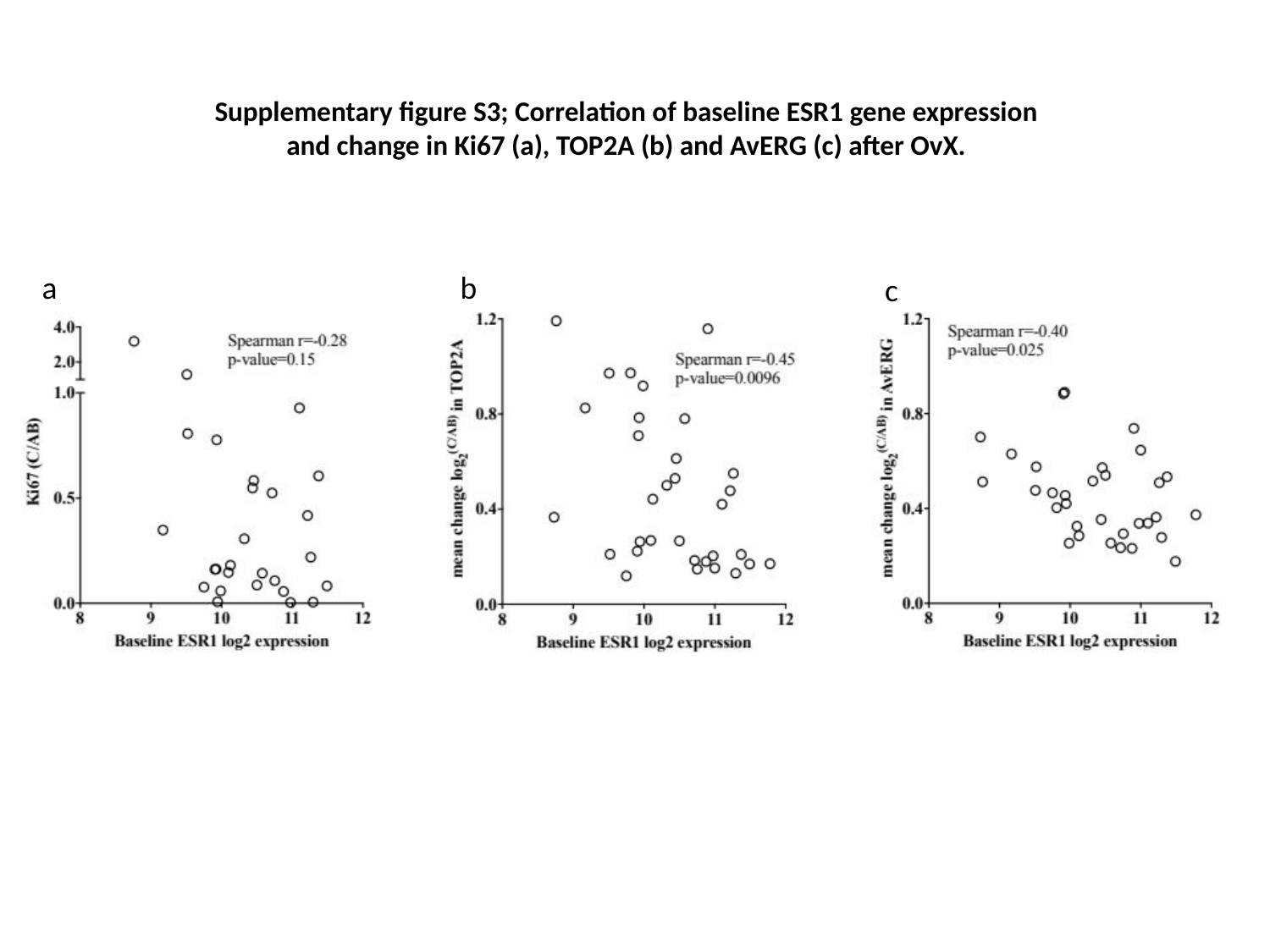

Supplementary figure S3; Correlation of baseline ESR1 gene expression and change in Ki67 (a), TOP2A (b) and AvERG (c) after OvX.
a
b
c
