# Supplementary material for: Molecular changes in premenopausal oestrogen receptor-positive primary breast cancer in Vietnamese women after oophorectomy
Source: NPJ Breast Cancer. 2017 Nov 27;3:47. doi: 10.1038/s41523-017-0049-z (PMC5703856; doi:10.1038/s41523-017-0049-z)
Supplement: Supplementary file 6 — Supplementary figure 4 [file 41523_2017_49_MOESM6_ESM.pptx]

## Slide 1
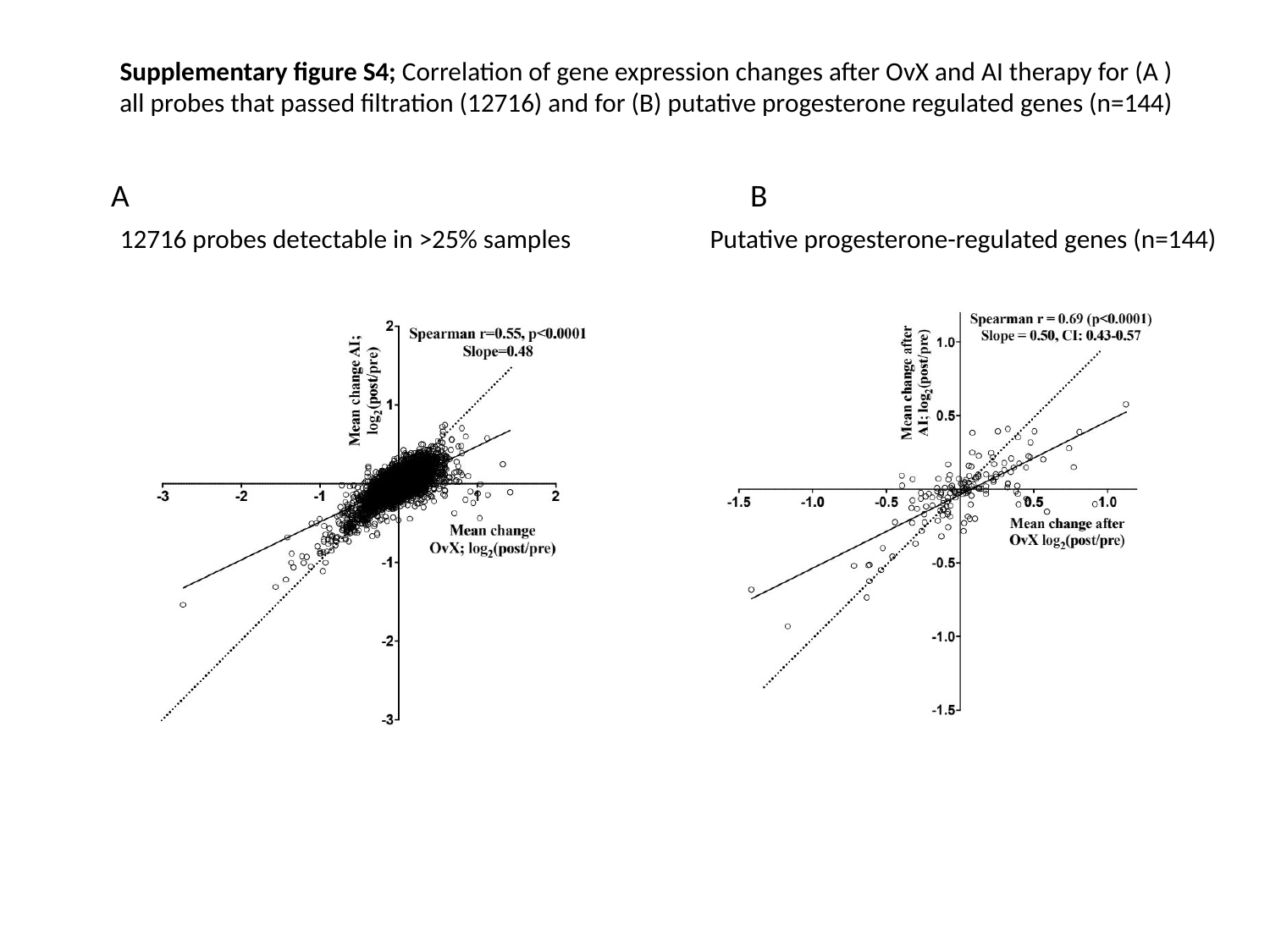

Supplementary figure S4; Correlation of gene expression changes after OvX and AI therapy for (A ) all probes that passed filtration (12716) and for (B) putative progesterone regulated genes (n=144)
A
B
12716 probes detectable in >25% samples
Putative progesterone-regulated genes (n=144)
